# Colorectal cancer and self-reported tooth agenesis

**DOI:** 10.1186/1897-4287-12-7

**Published:** 2014-03-10

**Authors:** Noralane M Lindor, Aung Ko Win, Steven Gallinger, Darshana Daftary, Stephen N Thibodeau, Renato Silva, Ariadne Letra

**Affiliations:** 1 Department of Health Sciences Research, Mayo Clinic, 13400 East Shea Blvd., Scottsdale, AZ 85259, USA; 2 Centre for Molecular, Environmental, Genetic, and Analytic Epidemiology, University of Melbourne, Melbourne, VIC, Australia; 3 Cancer Care Ontario, Toronto, ON, Canada; 4 Department of Laboratory Medicine and Pathology, Mayo Clinic, Rochester, MN, USA; 5 Department of Oral Biology and Center for Craniofacial and Dental Genetics, University of Pittsburgh, Pittsburgh, PA, USA; 6 Departments of Endodontics, School of Dentistry, and Pediatric Research Center, Medical School, University of Texas Health Science Center, Houston, TX, USA

**Keywords:** *AXIN2*, Colorectal neoplasia, Hypodontia, Oligodontia

## Abstract

**Background:**

Germline mutations in *APC* and *AXIN2* are both associated with colon neoplasia as well as anomalous dental development. We tested the hypothesis that congenitally missing teeth may occur more commonly in individuals diagnosed with colorectal cancer than in individuals without this diagnosis.

**Methods:**

Via a survey conducted on 1636 individuals with colorectal cancer (CRC) and 2788 individuals with no colorectal cancer from the Colon Cancer Family Registry, self-reported information on congenitally missing teeth was collected. The frequency of missing teeth between cases and controls was compared using Pearson’s chi-squared test or Fisher’s exact test.

**Results:**

4.8% of cases and 5.7% of controls reported having at least one missing tooth (p = 0.20). When we stratified by recruitment site, gender, and mutation status where available, frequency of missing teeth was not statistically significantly different between cases and controls.

**Conclusions:**

This study did not provide support for there being a general predisposition to missing teeth among a large cohort of CRC patients. The study neither addresses nor excludes the possibility, however, that individuals presenting with notable hypodontia/oligodontia might still have an increased risk for colorectal neoplasia.

## Background

Teeth are formed from neural crest-derived mesenchyme and epithelium of the first branchial arch and part of the frontonasal process [1,2]. The main proteins involved in development of dentition belong to one of five signaling pathways including Notch, bone morphogenetic protein, fibroblast growth factor, sonic hedgehog, and Wingless/integration (WNT).

A Finnish family was described in 2004 [3] in which a nonsense mutation in axis inhibition protein 2 (*AXIN2*) was found to co-segregate with an oligodontia (severe tooth agenesis) phenotype. Further characterization of the family identified adenomatous colorectal polyps and cancer as part of the phenotype. A second family, also in Finland, with the same *AXIN2* mutation was reported by Renkonen et al. [4] as part of their study of 29 polyposis families in which no adenomatous polyposis coli (*APC*) mutation was found. The *APC* gene, mutations of which cause Familial Adenomatous Polyposis, is a key component of the WNT-signaling pathway. Like *AXIN2*, *APC* mutations can be associated with dental anomalies including missing or supernumerary teeth [5].

The reported association between *AXIN2* variants, colorectal cancer, and tooth agenesis suggests that these conditions may be associated. However, no large-scale studies of colorectal cancer (CRC) patients have been conducted to assess a general association between colorectal neoplasia and missing teeth. The aim of this study was to determine if congenitally missing teeth may occur more commonly in individuals diagnosed with colorectal cancer than in individuals without this diagnosis. If so, then questioning people about missing teeth might help identify those at increased risk for colorectal cancer. In this study, we surveyed participants of the Colon Cancer Family Registry (C-CFR) for self-reported missing permanent teeth and compared findings with unaffected relatives and controls. We also assayed for the presence of *AXIN2* mutations in the only two probands presenting with colon cancer and tooth agenesis, and in family members with tooth agenesis but not cancer.

## Methods

Participants in this study were recruited through the C-CFR, a National Institutes of Health-supported consortium established to provide resources for the interdisciplinary study of the etiology of colon cancer and identification of at-risk populations who may benefit from translational research and current therapeutic strategies. The consortium consists of six research centers: Fred Hutchinson Cancer Research Center, Seattle, Washington; University of Hawaii Cancer Research Center, Honolulu, Hawaii; Mayo Clinic, Rochester, Minnesota; The University of Southern California Consortium, Los Angeles, California; Cancer Care Ontario, Ontario, Canada; and the University of Melbourne, Victoria, Australia. Detailed information about the C-CFR is described by Newcomb et al. [6]. This study included only CRC families from the Mayo Clinic and Toronto C-CFR sites, recruited both through the clinics and from population-based sources. After enrollment of the proband, recruitment invitation was extended to adult first- and second-degree relatives and in some families beyond that. Both Mayo Clinic and Cancer Care Ontario systematically oversampled for multiplex families and young-onset probands, but no information about dentition was known prior to enrollment. For C-CFR study participants with CRC, tumor characterization is routinely conducted to determine DNA mismatch repair status, and if abnormal, further testing is conducted for germline mutations in DNA mismatch repair genes [7]. Genetic testing for mutations in *MUTYH* is also standard for C-CFR probands [8]. Results of this molecular characterization were not used as inclusion or exclusion criteria for this study.

Standardized protocols were used to collect family history, risk factor information, and biospecimens. For this study, two questions were added to the 5- or 10-year follow-up survey. The first asked if the participant had any permanent teeth that never formed, not counting wisdom teeth. If they answered yes, the second question asked how many teeth were missing. Cases were defined as probands and relatives who had been diagnosed with colorectal cancer. Controls were defined as relatives and spouses who had never been diagnosed with any cancer. All study participants provided written informed consent and institutional review board approval was obtained at each center.

Five of the CRC cases with a missing tooth reported one or more relatives with missing teeth (none of those relatives had CRC). On the two Mayo probands and their five relatives, we performed direct sequencing of all *AXIN2* exons and exon-intron boundaries to look for mutations (DNA not available on the three multiplex Toronto families). Sequencing reactions were performed according to established protocols. Primer sequences and reaction conditions are available upon request.

The frequency of missing teeth between cases and controls were compared using Pearson’s chi-squared test or Fisher’s exact test. The comparisons were also made stratified by the recruitment site (Cancer Care Ontario, Mayo Clinic), gender (male, female), and germline mutation status (*MLH1, MSH2, MSH6, PMS2n MUTYH*). Note: the germline status of these genes was available in the C-CFR. These genes are not suspected of being involved with dental formation but were included in a stratified analysis just to be certain they were not contributing anything to the phenotype. Genetic testing for *APC* and *AXIN2* were not conducted systematically in the C-CFR so were not part of this study design nor stratified analyses. However, sequencing of *AXIN2* was conducted on two individuals who did have a colon cancer/missing teeth phenotype, and so this was reported, though it was not a primary aim of the study. All statistical analyses were conducted using STATA 12.1 [StataCorp. Stata Statistical Software: Release 12. College Station, TX: StataCorp LP; 2011].

## Results

Of a total of 1809 cases (1596 probands and 213 relatives), we excluded 173 (148 probands and 25 relatives) who did not provide missing teeth information. Of a total of 3005 controls (2737 relatives and 268 spouses), we excluded 217 (207 relatives and 10 spouses) who did not provide missing teeth information. The remaining 1636 cases (1448 probands and 188 relatives) and 2788 controls (2530 relatives and 258 spouses) entered into the analysis. Table 1 shows the baseline characteristics of the cases versus the controls.

**Table 1:** Self-reported missing teeth in study participants

		Cases*	**Controls**^**†**^
		**(n = 1636)**	**(n = 2788)**
**Missing teeth**			
	No	1558	2630
	Yes	78 (4.8%)	158 (5.7%)
**Number of missing teeth**			
	1	24	57
	2	22	54
	3	2	6
	4	7	9
	5	0	2
	≥6	1	6
	unknown	22	24

*Cases had diagnosis of colorectal cancer.

^†^Controls had no colorectal cancers.

We observed that 4.8% of cases and 5.7% of controls reported having at least one missing tooth (Table 1), and there was no statistical evidence of difference between them (p = 0.20). When we stratified by recruitment site, gender, and mutation status where available, frequency of missing teeth was not statistically significantly different between cases and controls. Women were reported to have more missing teeth than men in both cases (odds ratio [OR] = 2.21; 95% confidence interval [CI] = 1.30-3.93; p = 0.002) and controls (OR = 1.56; 95% CI = 1.09-2.27; p = 0.01; Table 2).

**Table 2:** Frequency of missing teeth between cases and controls

	**Cases**	**Controls**	**p value**
	**n/N (%)**	**n/N (%)**	
**Total**	78/1636 (4.8%)	158/2788 (5.7%)	0.20
**Recruitment site**			
Ontario	51/928 (5.5%)	88/1484 (5.9%)	0.66
Mayo Clinic	27/708 (3.8%)	70/1304 (5.4%)	0.12
**Gender**			
Male	20/695 (2.9%)	47/1094 (4.3%)	0.12
Female	58/941 (6.2%)	111/1694 (6.6%)	0.70
Mutation status*			
*MLH1*	1/63 (1.6%)	2/19 (10.5%)	0.13
*MSH2*	6/72 (8.3%)	3/35 (8.6%)	0.99
*MSH6*	0/8 (0%)	2/5 (40%)	0.13
*PMS2*	0/10 (0%)	0/6 (0%)	NA
All MMR genes	7/153 (4.6%)	7/65 (10.8%)	0.13
*MUTYH*	0/23 (0%)	1/24 (4.2%)	0.99

n = number of study participants with missing teeth.

N = total number of study participants.

*The germline status of these genes was available in the Colon Cancer Family Registry. These genes are not suspected of being involved with dental formation but were included in a stratified analysis just to be certain they were not contributing to the phenotype.

We looked to see if there were families in which multiple individuals reported missing teeth, and there were only a few. There were five CRC cases with a missing tooth reported in which one or more relatives had missing teeth (none of those relatives had CRC); eight of the CRC cases who themselves did not have missing teeth had two or more relatives with missing teeth; and of the CRC cases who did report one or more missing teeth, 24 had no other relatives surveyed in this study. All other probands had no reported missing teeth and 0-1 relatives with missing teeth.

Direct sequencing of the two Mayo probands and their five relatives with hypodontia revealed the presence of a common P50S missense variant in exon 10 of the *AXIN2* gene in one of the two probands with colon cancer and tooth agenesis, in four siblings with tooth agenesis but not colon cancer, and in one unaffected sibling (no colon cancer and no tooth agenesis). No *AXIN2* mutations were found in the second proband.

## Discussion

Prompted by the discovery of an association between colon polyps and cancer with tooth agenesis, several investigators have examined the potential for a colon neoplasia predisposition and WNT-signaling pathways genes from different perspectives. Peterlongo et al. [9] searched for *AXIN2* mutations in 82 familial (CRC families in which other syndromes had been ruled out. They found 29 DNA variants, none of which appeared likely to be pathogenic. Mostowska et al. [10] studied 55 Caucasians from Poland with missing teeth (nearly half had true oligodontia) and observed an increased risk of tooth agenesis in individuals carrying the *AXIN2* c.956 + 16G allele (OR = 2.94; 95% CI = 1.10-7.82; p = 0.03) and the c.2062 T allele (OR = 4.01; 95% CI = 1.56-10.30; p = 0.02). Lejeune et al. [11] studied 39 patients with multiple adenomas and no *APC* mutation nor DNA mismatch repair defect and found one person with two variants of uncertain significance in *AXIN2*. Callahan et al. [12] studied 167 individuals with tooth agenesis (majority with only 1-2 teeth missing) and found an association between missing incisors and one of three intragenic polymorphisms in the *AXIN2* gene. The most significant association was for a missense mutation in exon 10 (P50S, rs2240308) with a p-value of 0.037, and they concluded that their work provided further evidence that *AXIN2* contributes to tooth agenesis. This suggested relationship between *AXIN2* and tooth agenesis also led to findings that *AXIN2* gene variants may have a role in cleft lip/palate in multiple populations [13], with and without the presence of tooth agenesis as part of the clinical phenotype [14]. In a study of families with cleft lip and/or palate, a family history of cancer was reported more often than in control families with higher rates of specific cancer types including colon (p < 0.001) [15]. Most recently, Marvin et al. [16] reported a novel protein truncating *AXIN2* mutation (c.1989G > A; p.Try663X) that segregated in an autosomal-dominant pattern with colonic polyposis, gastric polyps, and a mild ectodermal dysplasia phenotype.

This current study was conducted to evaluate self-reported missing teeth as a potential marker for predisposition to CRC. We did not find that participants with CRC were any more likely to report missing teeth than their unaffected relatives or unrelated controls. To not form one or more of the normal 20 deciduous teeth and 32 permanent teeth is said to be one of the most common developmental anomalies in humans.

Approximately 20% of humans will have at least one missing third molar (“wisdom teeth”) and 3-10% have failure of formation for one or more of the other permanent teeth (reviewed in Nieminen 2009 reference). Both our CRC cases and the unaffected control group reported frequencies of missing teeth consistent with this range. The term hypodontia is a term used to describe only a few missing teeth, while oligodontia refers to a more severe anomaly with six or more missing teeth. True oligodontia is quite rare, affecting 0.1-0.2% of the population [17]. Seven of 4244 (0.16%) individuals in this study reported true oligodontia, also in keeping with the literature; thus, the self-reported nature of this study is a limitation but the reported values are consistent with other studies. In general, failure to form one or more permanent teeth (excluding third molars) is reported to be about twice as common in females as in males, and again, our data reflect that trend.

Overall, this study did not provide support for there being a general predisposition to missing teeth among a large cohort of CRC patients. It does not, however, rule out the likelihood that a small percentage of individuals may have germline variants in genes that predisposed them to both CRC and missing teeth. It may be that if a population of people with oligodontia were surveyed for cancer risks, one might still find that this is a marker for colorectal neoplasia but that oligodontia is so uncommon as to not be appreciable in a study of the size we conducted.

Of note, tooth agenesis is a common, multifactorial birth defect and can be sporadic or familial (inherited as dominant, recessive, or X-linked), and present variable penetrance in affected families [17]. It also presents variable prevalence rates among different ethnic backgrounds; therefore, the use of family-based studies may allow detection of segregation between different phenotypes and improve the likelihood of gene discovery in affected families.

*AXIN2* mutations have been regarded as causative for the segregation of CRC and oligodontia in a Finnish family [3]. In the present study, we sequenced the *AXIN2* gene locus in two probands presenting colon cancer and tooth agenesis (although the milder form, hypodontia) and having relatives with tooth agenesis. We found a common missense variant (P50S, rs2240308; minor allele frequency approximately 0.35 in North America) in one of the probands. Five of this proband’s siblings including an unaffected sister (without tooth agenesis) also presented the variant. The *AXIN2* P50S variant is unlikely related to the familial tooth agenesis or the colon cancer phenotype in this family. Additional, and yet possibly unknown genes, may be contributing to the segregation of these two different clinical phenotypes. Additional studies are underway to molecularly characterize the relevant genes in this population, with special attention to those few families that did report familial hypo- or oligodontia.

## Conclusion

This study did not provide support for there being a general predisposition to missing teeth among a large cohort of CRC patients. The study does not exclude the possibility, however, that individuals presenting with notable hypodontia/oligodontia might still have an increased risk for colorectal neoplasia.

## Abbreviations

APC: Adenomatous polyposis coli; CI: Confidence interval; C-CFR: Colon cancer family registry; CRC: Colorectal cancer; OR: Odds ratio; WNT: Wingless/integration.

## Competing interests

The authors declare that they have no competing interests.

## Authors’ contributions

NML conceived of the study. SG, NML, and DD collected the data. NML and AKW performed the data analysis. AL and RS conducted the sequencing of the AXIN2 gene. All authors read, critically reviewed, and approved the final manuscript.
